# Miniature Negative Pressure Dressings on Forearm Donor Sites after Radial Forearm Flap Harvest

**DOI:** 10.1097/GOX.0000000000001838

**Published:** 2018-06-15

**Authors:** Edward Ray, Stuart L. Mitchell, Peter G. Cordeiro

**Affiliations:** From the Plastic and Reconstructive Surgery Service, Memorial Sloan Kettering Cancer Center, New York, N.Y.

## Abstract

Management of radial forearm donor sites following free flap reconstruction of head and neck tumor defects typically requires split-thickness skin grafting. Tendon exposure and delayed healing are occasional problems associated with skin grafting over the distal forearm musculature. Others have reported the use of negative pressure wound therapy (NPWT) devices to bolster split-thickness grafts and improve graft take. Although this approach works well, these devices are not always approved by third-party payers for outpatient use, requiring patients to remain in the hospital or have these devices removed before discharge. The authors report outcomes using a miniaturized NPWT device to bolster the skin graft on radial forearm free flap donor sites in 10 consecutive patients compared with 10 consecutive control patients managed with traditional bolster dressings. The 2 groups of patients were similar in terms of demographics, indication for reconstruction, and size of donor site. There was less skin graft loss and a lower rate of tendon exposure and infection in the miniaturized NPWT device group, though these results did not reach statistical significance. Recently developed miniaturized, single-use NPWT devices can be reliably used as bolsters for skin grafts with some notable advantages over reusable NPWT devices.

## INTRODUCTION

Flap donor site management poses a secondary challenge in head and neck reconstruction following oncologic extirpation. Frequently used free flaps include the anterolateral thigh flap, the radial forearm flap, and the free fibula osseous, or osteocutaneous flap, each of which may leave a donor site that cannot be closed without a skin graft. The morbidity of failed skin grafts on donor sites includes tendon exposure, prolonged wound healing, and infection. Ensuring maximal skin graft take in such defects is of great importance, and others have demonstrated the benefit of negative pressure wound therapy (NPWT) for this purpose.^1–8^ Miniaturized, one-time use NPWT devices have recently become available.^9^ We present our experience with such a device used as a bolster for radial forearm free flap donor site skin grafts at the time of oncologic head and neck reconstruction.

## METHODS

**Fig. 1.**
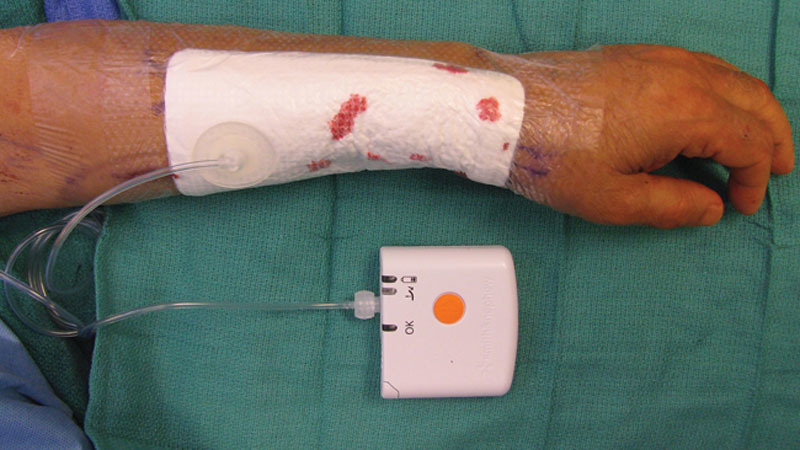
PICO device in place over skin graft on forearm donor site.

**Fig. 2.**
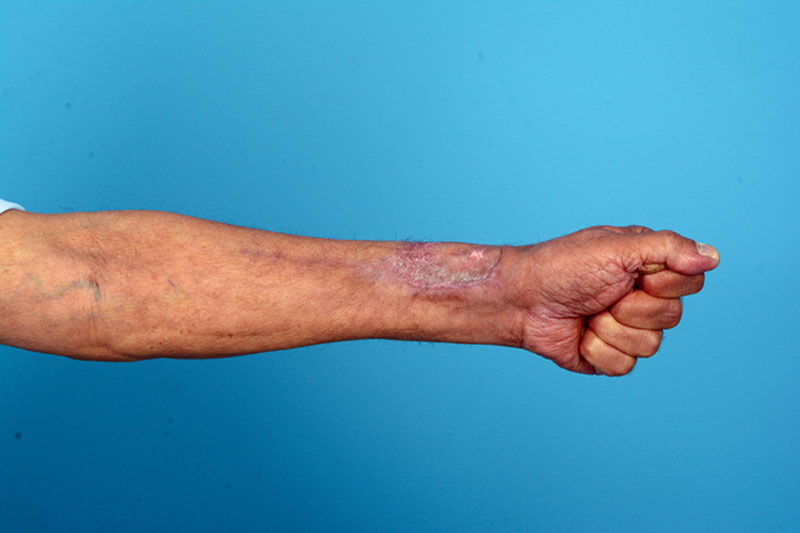
Skin-grafted radial forearm flap donor site, 2 months after surgery.

We retrospectively reviewed the records for 20 consecutive patients who underwent head and neck tumor removal requiring a radial forearm free flap with a donor site that could not be closed primarily. All reconstructive procedures were performed by a single surgeon (P.C.). Split-thickness skin grafts (13/1000ths of an inch thick) obtained from the thigh were placed as pie-crusted sheet grafts over the flap donor sites. The grafts were sutured to the surrounding skin edges with 4-0 chromic gut suture. A contact layer of Adaptic (Acelity, San Antonio, Tex.) was applied over the skin graft, followed by either standard bolster dressing (control group, n = 10) or the proprietary absorptive/evaporative dressing supplied with the PICO (Smith & Nephew, Hull, United Kingdom) NPWT device (NPWT group, n = 10). Once attached and activated, the NPWT device maintained 80 mm Hg continuous negative pressure within the dressing (Fig. 1). To minimize skin graft shearing due to tendon excursion, the donor forearm was temporarily immobilized using a plaster volar wrist splint with thumb spica (both groups). On postoperative day 5, the splint was replaced with a removable wrist splint. The dressing was removed after 1 week to allow for skin graft assessment. The grafts were then redressed daily with a nonadherent Xeroform (DeRoyal Industries, Inc., Powell, Tenn.) petrolatum gauze layer, followed by a gauze wrap. Splinting continued until the graft adhered sufficiently and immobilization was no longer needed (Fig. 2).

Patient characteristics and outcomes were analyzed using Fisher’s exact test for categorical parameters and Student’s *t*-test for continuous parameters. A *p*-value less than 0.05 was considered statistically significant.

## RESULTS

Twenty consecutive patients (Table 1) over a 24-month period were reconstructed for cancers of the head and neck using radial forearm free flaps: 10 were treated with a standard bolster dressing (control group) and 10 with the miniaturized NPWT device (NPWT group). There were no statistically significant differences observed in patient characteristics or outcomes (Table 2).

**Table 1.**
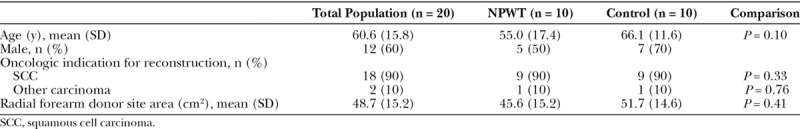
Patient Characteristics

**Table 2.**
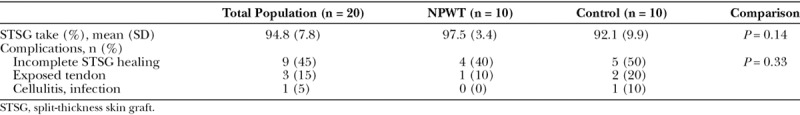
Patient Outcomes and Complications

### Control Group

The mean age was 66.1 years (range, 50–85 years); 7 were men (70%). Nine patients had squamous cell carcinomas, and 1 had salivary gland carcinoma. The area of the radial forearm donor site defects ranged from 28 to 80 cm^2^. Five patients had at least some degree of graft loss, with mean area of 6.2 cm^2^ (range, 4–10.5 cm^2^), and 2 had exposed tendon in the wound bed. One patient had a local wound infection.

### Negative-pressure Wound Therapy Group

The mean age was 55 years (range, 28–80 years); 5 were men (50%). Similar to the control group, 9 patients had squamous cell carcinomas, but 1 had an invasive thyroid carcinoma requiring laryngectomy and partial hypo-pharyngectomy. The forearm donor site defects ranged in size from 28 to 80 cm^2^. Six skin grafts healed without any difficulty. Four required local wound care for areas of skin graft loss (mean, 2.8 cm^2^; range, 2–4.8 cm^2^), of which 1 had exposed flexor carpi radialis tendon in the wound bed. All 4 patients healed secondarily within 2 months. There were no other complications with the donor sites.

## DISCUSSION

NPWT has a wide range of applications in postoperative wound management, including the protection of healing skin grafts.^1–8,10^ NPWT effectively bolsters skin grafts, isolating them from outside contamination, removing excess exudate, preventing desiccation, and minimizing separation of the graft from the wound bed that might lead to seroma formation.^9^ NPWT typically includes portable devices that are rented by health care facilities or individuals in the outpatient setting, pending the approval of third-party payers. A single-use, super-miniaturized device with no per diem cost or need for outpatient monitoring by specialty nursing services and that can be used continuously over a 7-day period is greatly useful to patients with skin grafts following reconstruction. Traditional skin graft bolsters are typically removed within a week of grafting to assess the graft and prevent maceration or desiccation. We have found that use of the miniaturized NPWT device, at $218 per unit, is effective from a cost and convenience standpoint. Graft healing appears to be equivalent to that seen after use of larger, costlier systems and traditional bolsters, as demonstrated in this study. Anecdotally, we have observed that skin grafts treated with NPWT have better aesthetics in the early stages of healing. Tie-over bolsters traditionally used to stabilize skin grafts may leave a deeper contour defect than typically seen with NPWT devices, in our experience. Randomized, controlled studies comparing outcomes and cost between traditional dressings and single-use, portable NPWT dressings are needed to make further evidence-based conclusions.

## CONCLUSIONS

The single-use NPWT device represents a new method to simplify bolstering of skin grafts over sizable donor sites following the harvest of free flaps for head and neck reconstruction. This miniaturized NPWT device appears to work as well as traditional bolsters and larger, commercially available NPWT systems while possessing notable advantages, including smaller size, disposability, ease of application, and reliability.^9^ Patients may be discharged from the hospital before removal of this device, obviating the need for insurance approval and expensive NPWT equipment rental. Miniature, disposable NPWT dressings effectively manage wound exudate via an evaporative process, avoiding maceration while promoting graft adherence.

## PATIENT CONSENT

Patients provided written consent for the use of their images.

## References

[1] Avery C Pereira J Moody A Negative pressure wound dressing of the radial forearm donor site. Int J Oral Maxillofac Surg. 2000;29:198 200. 10970082

[2] Trop M Schintler M Urban E Are 1:4 mesh and donor site contraindications for vacuum-assisted closure device? J Trauma. 2006;61:1267 1270. 17099542 10.1097/01.ta.0000241149.20000.55

[3] Andrews BT Smith RB Chang KE Management of the radial forearm free flap donor site with the vacuum-assisted closure (VAC) system. Laryngoscope. 2006;116:1918 1922. 17003705 10.1097/01.mlg.0000235935.07261.98

[4] Ho MW Rogers SN Brown JS Prospective evaluation of a negative pressure dressing system in the management of the fibula free flap donor site: a comparative analysis. JAMA Otolaryngol Head Neck Surg. 2013;139:1048 1053. 24008650 10.1001/jamaoto.2013.4544

[5] Bach CA Guilleré L Yildiz S Comparison of negative pressure wound therapy and conventional dressing methods for fibula free flap donor site management in patients with head and neck cancer. Head Neck. 2016;38:696 699. 25522136 10.1002/hed.23952

[6] Vidrine DM Kaler S Rosenthal EL A comparison of negative-pressure dressings versus Bolster and splinting of the radial forearm donor site. Otolaryngol Head Neck Surg. 2005;133:403 406. 16143190 10.1016/j.otohns.2005.04.028

[7] Sposato G Molea G Di Caprio G Ambulant vacuum-assisted closure of skin-graft dressing in the lower limbs using a portable mini-VAC device. Br J Plast Surg. 2001;54:235 237. 11254417 10.1054/bjps.2000.3537

[8] Chio EG Agrawal A A randomized, prospective, controlled study of forearm donor site healing when using a vacuum dressing. Otolaryngol Head Neck Surg. 2010;142:174 178. 20115970 10.1016/j.otohns.2009.11.003

[9] Payne C Edwards D Application of the single use negative pressure wound therapy device (PICO) on a heterogeneous group of surgical and traumatic wounds. Eplasty. 2014;14:e20. 24917894 PMC4006427

[10] Schmedes GW Banks CA Malin BT Massive flap donor sites and the role of negative pressure wound therapy. Otolaryngol Head Neck Surg. 2012;147:1049 1053. 22949007 10.1177/0194599812459015

